# Draft Genome Sequence of *Rhizobium* sp. GHKF11, Isolated from Farmland Soil in Pecan Grove, Texas

**DOI:** 10.1128/genomeA.00682-16

**Published:** 2016-07-21

**Authors:** Rupa Iyer, Ashish Damania

**Affiliations:** a Center for Life Sciences Technology, College of Technology, University of Houston, Houston, Texas, USA; b Department of Pediatrics-Tropical Medicine, Baylor College of Medicine, Houston, Texas, USA

## Abstract

*Rhizobium* sp. GHKF11 is an organophosphate-degrading bacterial strain that was isolated from farmland soil in Pecan Grove, Texas, USA. In addition to a capacity for pesticide degradation, GHKF11 shares conserved traits with other *Rhizobium* spp., including heavy metal resistance and transport genes that may have significant agricultural biotechnology applications.

## GENOME ANNOUNCEMENT

Soil bacteria that live within the rhizosphere of plants often form symbiotic relationships with their plant hosts, significantly enhancing their nutrient uptake, fixing nitrogen, and promoting plant growth. Of these soil bacteria, strains that are resistant to the toxicological effects of common pesticides and heavy metals and aid in the soil detoxification of these compounds are particularly desirable to agricultural biotechnology (1, 2). Here, we report a draft genome sequence of an organophosphate-degrading strain of a *Rhizobium* sp., isolated from farmland soil in Pecan Grove, Texas, USA, through an environmental sampling research module undertaken by University of Houston biotechnology undergraduates (3). This strain, designated GHKF11, is related to *Rhizobium* sp. UR51a and shares many of its plant-promoting characteristics, including genes involved in nodulation, nitrogen fixation, and the production of siderophores (4). GHKF11 also possesses a battery of resistance genes against metals and metalloids, such as copper, cobalt, nickel, and arsenic. With the addition of its phosphotriesterase activity against organophosphates, the GHKF11 strain shows considerable potential for aiding in the remediation of both pesticide- and metal-contaminated soils.

The genome sequencing of GHKF11 was performed through Illumina MiSeq paired-end sequencing with a final sequencing coverage of 326.38×. Sequence reads were checked for quality using FastQC (5) and filtered using BBTools (6) with a minimum Phred score of 20. Paired-end reads were assembled into 61 contigs with the SPAdes version 3.7 program (7). Preliminary reference-based annotation using PATRIC (8) web resources was carried out to identify conserved pathways. Final *de novo* annotation was performed with Prokka (9) and the NCBI Prokaryotic Genome Automatic Annotation Pipeline (http://www.ncbi.nlm.nih.gov/genome/annotation_prok). The metabolic pathways of aromatic and heterocyclic compounds were examined through KEGG databases (10). This draft genome of strain GHKF11 comprises a total of 5,290,899 bp encoding for 4,899 protein-coding sequences, of which 97 are pseudogenes, 1,059 are annotated as hypothetical proteins, and 3,840 form known functional proteins. The genome has a GC content of 59.12% and contains 4 rRNA (three complete, two partial), 50 tRNA, and 4 ncRNA loci.

### Nucleotide sequence accession numbers.

The *Rhizobium* sp. GHKF11 whole-genome shotgun project has the project accession number LVFG00000000. This version of the project (01) has the accession number LVFG01000000, and consists of sequences LVFG01000001 to LVFG0100061.
